# Key attributes of child psychiatry access programs

**DOI:** 10.3389/frcha.2023.1244671

**Published:** 2023-07-25

**Authors:** Yael Dvir, John H. Straus, Barry Sarvet, Nancy Byatt

**Affiliations:** ^1^ Division of Child and Adolescent Psychiatry, Department of Psychiatry, University of Massachusetts T.H. Chan Medical School, UMass Memorial Health, Worcester, MA, United States; ^2^ Massachusetts Child Psychiatry Access Program/Carelon Behavioral Health, Boston, MA, United States; ^3^ Department of Psychiatry, University of Massachusetts T.H. Chan Medical School-Baystate, Springfield, MA, United States; ^4^ Lifeline for Families Division, Department of Psychiatry, University of Massachusetts T.H. Chan Medical School, UMass Memorial Health, Worcester, MA, United States

**Keywords:** access programs, behavioral health, primary care clinicians, program evaluation, consultation

## Abstract

The gap between the need for and the availability of pediatric mental health providers is well documented. One solution is regional/state Child Psychiatry Access Programs (CPAPs), which aid in the assessment and management of youth with behavioral health (BH) concerns by providing consultation to Pediatric Primary Care Clinicians. Our authorship team and the National Network of Child Psychiatry Access Programs (NNCPAP) board worked to describe operational definitions for CPAPs elements and related outcome monitoring processes and data systems. CPAP elements include regional child psychiatry availability by phone; real time phone availability; referral and resource assistance; and, expedited face-to-face psychiatric evaluation. Defining a child psychiatry consultation program as a CPAP and describing key attributes for CPAPs is an important step in facilitating implementation of the model and advancing research into its effectiveness.

## Introduction

The nationwide gap between the need for pediatric mental health providers and their availability in the United States (US) is well documented: only 20% of youth with a mental health disorder receive care from a specialized Behavioral Health (BH) provider (1). While only one in three pediatricians report sufficient training to diagnose and treat children with behavioral disorders, pediatric primary care clinicians prescribe most psychiatric medications for children in outpatient settings (1). BH integration into primary care is a response important to the challenge of inadequate child BH workforce capacity (2). One approach to integrating behavioral health care into pediatric primary care is statewide Child Psychiatry Access Program model (CPAPs). CPAPs increase access to child mental health care by providing a system of regional children's BH consultation teams that help pediatric primary care clinicians and practices manage the BH of pediatric patients (4). The first CPAP was established in Massachusetts in 2004 to aid pediatric primary care clinicians in providing psychiatric treatment to youth through consultation and referral services (3). In regions with a statewide CPAP, children are significantly more likely to receive mental health services (5). The Health Resources and Services Administration (HRSA) funding for Pediatric Mental Health Care Access (PMHCA) in the US 2021 and 2022 (6) expanded pediatric BH integration and led to similar programs across and beyond the US. There are now 46 CPAPs under development and/or existing in 46 states, as well programs in the District of Columbia, two tribal communities and four US territories, and internationally in British Columbia, Canada, and Australia. As a model, CPAPs are designed to assist primary care clinicians with managing children with mild to moderate psychiatric illness by providing real time phone consultation, referral and resource assistance and expedited face-to-face psychiatric evaluation.

A systematic review by Bettencourt and colleagues of methods used to evaluate CPAPs published in 2020, reviewed 29 publications evaluating 13 programs in 11 states, focusing primarily on program utilization and provider satisfaction, practices, and self-efficacy. With respect to outcomes for patients and families, the authors noted that the available publications analyzed access to treatment rather than behavioral health outcomes because the studies being conducted where largely descriptive rather than experimental (7). Nevertheless, key findings from these studies suggest that primary care clinicians adopt the model, are satisfied with direct consultation for diagnostic assistance and medication related questions, and that their ability to manage more complex cases increases with time (7). Additionally, most studies evaluated the consultation/evaluation and referral service rather than the education/training component, making it difficult to draw conclusions about the different elements of the model. Likewise, there has been limited focus on patient outcomes (7).

A major barrier to the study of these programs is the absence of a consensus description and set of criteria defining the CPAP model. The National Network of Child Psychiatry Access Programs (NNCPAP) is a nation-wide association established in 2011 to support existing and emerging CPAPs by creating of a data base and toolkit for new programs, and collaboration on evaluation and research (8). In this perspective article, we outline CPAP operational definitions that were developed based on the existing literature and expert consensus. Experts included members of the NNCAP board and leaders of CPAP programs. The definitions were developed based on an initial consensus then refined based on interactive feedback from these experts.

## Definition and core elements

### Definition

Child Psychiatry Access Programs (CPAPs) are a model of care wherein pediatric primary care clinicians receive training and support in treating their patients with regard to mental health and substance use disorders via in-person, phone, or online case-based consultation from off-site mental health professionals.

### Primary goal of CPAPs

Increase access to pediatric mental health and substance use services by providing consultation and training to pediatric primary care clinicians to increase their comfort and skills in managing mild to moderate mental health and substance use disorders.

### CPAP elements

1. Provision of on-demand telephonic consultation by child and adolescent psychiatrists and optionally other licensed children's mental health professionals to pediatric primary care clinicians and integrated behavioral health clinicians in the pediatric primary care practices in/for a defined region/population regarding diagnosis and management of mental health and substance use disorders.
2. Real time response (calls returned within 30 min when possible, or at least within same workday). Real time response can also be available through asynchronous communication (email, secure messaging).
3. Provision of referrals and resource navigation.
4. Availability of expedited in-person or virtual psychiatric evaluation when indicated.
5. Formal continuing education sessions for pediatric primary care clinicians and assistance with practice transformation to integrate behavior health. Educational tools may include practice guidelines, website, webinars, and newsletters.

### CPAP outcome and performance monitoring

CPAPs use a data system to monitor program performance and outcomes. Usage is monitored with an encounter database which captures demographic information about the patient served, the provider requesting help, the type of presenting mental health problem, the nature of the presenting need or question, and the type of service provided to the pediatric primary care clinicians and/or patient. In addition, a web-based database includes number of practices and/or pediatric primary care clinicians enrolled and annual utilization; pediatric primary care clinicians' satisfaction; and pediatric primary care clinicians’ comfort and confidence with managing mental health concerns (change from baseline). Additional elements may include measures of provider practice change, including the use of screening, practice guidelines, measurement-based practice, and total practice BH outcomes; patient outcomes; and family member satisfaction with services. Performance measures include the degree to which the program reaches the entire geographic region population (percent of practices enrolled and utilizing).

## Discussion and next steps

CPAPs are a model for providing pediatric BH care created in response to unmet BH needs among children and adolescents, resource and workforce gaps, and deficits in graduate medical education training for pediatric primary care clinicians. The CPAP has grown in recent years and continues to grow. While there are currently CPAP versions in multiple states as well as international programs, there is limited multistate or nationwide research and no formal operational definitions of a CPAP. Taken together, this suggests that policy makers and funders are moving forward with supporting the CPAP model as a solution to pediatric behavioral health care access, without the benefit of clear data on the effectiveness of the model.

Better understanding of the different components of each program, and the establishment of consensus criteria for the model is an important first step in the development of measures that could facilitate outcome evaluation on a larger scale (9). In this paper, we present operational definitions of program elements, outcome measurement processes and database systems as a first step toward to facilitating such evaluation.

## Data Availability

The original contributions presented in the study are included in the article/Supplementary Material, further inquiries can be directed to the corresponding author.
